# Genome-wide analysis suggests high level of microsynteny and purifying selection affect the evolution of *EIN3/EIL* family in Rosaceae

**DOI:** 10.7717/peerj.3400

**Published:** 2017-05-31

**Authors:** Yunpeng Cao, Yahui Han, Dandan Meng, Dahui Li, Qing Jin, Yi Lin, Yongping Cai

**Affiliations:** 1 College of Life Sciences, Anhui Agricultural University, Hefei, China; 2 State Key Laboratory of Tea Plant Biology and Utilization, Anhui Agricultural University, Hefei, China

**Keywords:** Rosaceae, *EIN3/EIL*, Microsynteny, Purifying selection, qRT-PCR

## Abstract

The ethylene-insensitive3/ethylene-insensitive3-like (*EIN3/EIL*) proteins are a type of nuclear-localized protein with DNA-binding activity in plants. Although the *EIN3/EIL* gene family has been studied in several plant species, little is known about comprehensive study of the *EIN3/EIL* gene family in Rosaceae. In this study, ten, five, four, and five *EIN3/EIL* genes were identified in the genomes of pear (*Pyrus bretschneideri*), mei (*Prunus mume*), peach (*Prunus persica*) and strawberry (*Fragaria vesca*), respectively. Twenty-eight chromosomal segments of *EIL/EIN3* gene family were found in four Rosaceae species, and these segments could form seven orthologous or paralogous groups based on interspecies or intraspecies gene colinearity (microsynteny) analysis. Moreover, the highly conserved regions of microsynteny were found in four Rosaceae species. Subsequently it was found that both whole genome duplication and tandem duplication events significantly contributed to the *EIL/EIN3* gene family expansion. Gene expression analysis of the *EIL/EIN3* genes in the pear revealed subfunctionalization for several *PbEIL* genes derived from whole genome duplication. It is noteworthy that according to environmental selection pressure analysis, the strong purifying selection should dominate the maintenance of the *EIL/EIN3* gene family in four Rosaceae species. These results provided useful information on Rosaceae *EIL/EIN3* genes, as well as insights into the evolution of this gene family in four Rosaceae species. Furthermore, high level of microsynteny in the four Rosaceae plants suggested that a large-scale genome duplication event in the *EIL/EIN3* gene family was predated to speciation.

## Introduction

Rosaceae species such as pear (*Pyrus bretschneideri*), mei (*Prunus mume*), peach (*Prunus persica*) and strawberry (*Fragaria vesca*) are important perennial trees cultivated for the commercial production of fruits available worldwide. According to previous studies, the genomes of strawberry (*X* = 7), mei (*X* = 8), peach (*X* = 8), and pear (*X* = 17) shared an ancestor, which had nine pairs of chromosomes (Shulaev et al., 2011; Verde et al., 2013; Wu et al., 2012; Zhang et al., 2012). Recently, the researchers confirmed that chromosome inversions, fusions, and translocations played an important role in the evolution of the Rosaceae genome (Illa et al., 2011). Some extant “diploid” species of Rosaceae family are originated from their polyploid ancestors, others are actually thought to be true polyploids (Wendel, 2000). These studies indicate that the diploid species in Rosaceae have evolved with a complex history. There are several gene families which share highly conserved genome sequences with each other among the related species of family Rosaceae, as well as other taxonomic families. In this study, the *EIL/EIN3* gene family was selected to investigate the specific evolutionary relationships among the related species of family Rosaceae.

The *EIN3/EIL* gene family is a relatively small one in higher plants. Some *EIN3/EIL* genes have been isolated from *Arabidopsis thaliana* (Chao et al., 1997), tobacco (Kosugi & Ohashi, 2000; Rieu, Mariani & Weterings, 2003), banana (Jourda et al., 2014), tomato (Tieman et al., 2001; Yokotani et al., 2003) and rice (Hiraga et al., 2009; Mao et al., 2006). These plant-specific EIN3/EIL proteins are located in the nuclei, with the highly conserved amino acid sequences at the N-termini, including several important structural features, such as acidic amino acid regions, proline-rich regions and 5-basic amino acid clusters (BD I–V) (Chao et al., 1997). Compared to the N-terminal sequences, the conservation of their C-termini is lower. For example, it was found that although asparagine-enriched regions or glutamine-enriched regions were commonly distributed within the C-terminal sequences of EIN3/EILs in Arabidopsis, carnation and mung beans (Chao et al., 1997; Lee & Kim, 2003; Waki et al., 2001), they did not widely exist in other EIN3/EIL members, such as tobacco NtEILs (Rieu, Mariani & Weterings, 2003).

Functions of the *EIN3/EIL* gene family have been studied in several plants, such as *Hevea brasiliensis* (Yang et al., 2015) and tomato (Tieman et al., 2001; Yokotani et al., 2003). Recently, research on the application of comparative genome in the analysis of evolution and function of gene family have been reported. For example, based on the comparative genomic analysis, Wang et al. (2015) explored the evolution and functional differences of WRKY type-III transcription factor family of poplar, grape, Arabidopsis and rice. Jing et al. (2016) explored the evolution of WRKY I subfamily in Gramineae. However, there is still lack of specific evolutionary relationships of the *EIN3/EIL* gene family in Rosaceae. To address this question, the evolutionary relationships and gene duplication events of *EIN3/EIL* genes from Rosaceae species, including pear (*Pyrus bretschneideri*), mei (*Prunus mume*), peach (*Prunus persica*) and strawberry (*Fragaria vesca*), were analyzed, based on their phylogenetic relationships, microsynteny and environmental selection pressures analysis. In addition, the expression patterns of pear *EIN3/EIL* genes were investigated on a variety of organs/tissues including fruits at several developmental stages. The results obtained from this study provided valuable information about *EIN3/EIL* genes that will aid future functional research involved in many important biological processes of this important gene family in flowering plants, especially in the pear.

## Materials and Methods

### Sequence identification and collection

The genome data of four Rosaceae species were obtained from their respective genome sequence websites: *Pyrus bretschneideri* from the GigaDB database (http://gigadb.org/site/index); *Prunus mume* from the Genome Database for Rosaceae (http://www.rosaceae.org/); *Prunus persica* from the Phytozome database (https://phytozome.jgi.doe.gov/pz/portal.html) and *Fragaria vesca* from the Joint Genome Institute (http://www.jgi.doe.gov/). The Hidden Markov Model (HMM) profiles of EIN3/EIL domain (PF04873) (Chang & Shockey, 1999; Chao et al., 1997) were obtained from the Pfam database (http://pfam.xfam.org) (Finn et al., 2006). The EIN3/EIL domain was used as query sequences to identify *EIN3/EIL* genes in four Rosaceae species by using DNAtools software (*E*-value < 0.001). To verify the *EIN3/EIL* genes in four Rosaceae genomes, all putative proteins were validated by searching for the EIN3/EIL domain using the InterPro online tool (http://www.ebi.ac.uk/interpro/) (Zdobnov & Apweiler, 2001) and SMART database (http://smart.embl-heidelberg.de/) (Letunic, Doerks & Bork, 2012). In our study, only the EIN3/EIL domain-containing sequences were retained.

### Chromosomal location of *EIN3/EIL* genes

The genome annotation information was collected from GigaDB database (http://gigadb.org/site/index), Genome Database for Rosaceae (http://www.rosaceae.org/), Phytozome database (https://phytozome.jgi.doe.gov/pz/portal.html) and Joint Genome Institude (http://www.jgi.doe.gov/), respectively. Subsequently, the MapInspect software (http://mapinspect.software.informer.com/) was used for data visualization.

### Gene structure and motif analysis

The exon-intron structure of each *EIN3/EIL* gene was determined by alignment of its CDS and genomic DNA sequence. Then a diagram was constructed using the Structure Display Server website (Hu et al., 2014). Subsequently, the Online MEME server was used to screen the conserved motifs encoded by *EIN3/EIL* genes. Additionally, the Pfam website (Punta et al., 2012) and SMART tools (Letunic, Doerks & Bork, 2012) were used to annotate these structural motifs.

### Phylogenetic analysis of *EIN3/EIL* genes

EIN3/EIL sequences were aligned using ClustalX version 1.83 (Thompson et al., 1997) and evolutionary relationships were inferred by analyzing an unrooted phylogenetic tree using MEGA 5 and neighbor-joining (NJ) method (Tamura et al., 2011) with the following parameters: poisson correction, pairwise deletion and 1,000 bootstrap replicate.

### Microsynteny analysis

In order to reveal the sequence features of the *EIN3/EIL* gene-containing regions, microsynteny analysis was performed across the four Rosaceae species using MCScanx (Multiple Collinearity Scan toolkit) (Wang et al., 2012) with the gene identifier file, the gene list file and the coding sequence file. Subsequently, a syntenic block was defined as a region containing three or more conserved homologs which were located within 100-kb downstream and upstream of protein-coding sequences.

### Environmental selection pressure analysis

The nucleotide coding sequences from segmentally duplicated pairs were aligned by Clustal X (Thompson et al., 1997). Then DnaSP (version 5.10) was used to calculate the nonsynonymous (Ka) and synonymous (Ks) substitution rates of the homologues (Librado & Rozas, 2009). For each pair of duplicated regions, we estimated the mean Ks values of the flanking conserved genes for individual homologs. To further understand the selective pressure experienced by *EIN3/EIL* genes, Ka, Ks and Ka/Ks ratios were estimated using sliding window (with parameters: window size, 150 bp; step size, 9 bp) over the entire aligned length (Cao et al., 2016a; Han et al., 2016).

### *EIN3/EIL* gene expression analysis in pear different tissues

To verify the expression patterns of *EIN3/EIL* genes, qRT-PCR analysis was carried out. The first-strand cDNA was synthesized with Oligo18dT primer (Table S1) by using M-MLV reverse transcriptase (TakaRa, Japan) following the manufacture introduction. The TransStart Tip Green qPCR SuperMix (TransGen Biotech, China) with SYBR Green I as the fluorescent dye was used for the qPCR, employing a Bio-rad CFX96 Touch™Deep Well Real-Time PCR Detection system (BioRad, USA). The transcript level relative to the Pyrus tubulin gene (Wu et al., 2012) was estimated according to a previous workflow (Cao et al., 2016a; Cao et al., 2016b). For each sample, three replicates were set up in parallel experiments.

## Results and Discussion

### Identification of *EIN3/EIL* genes in Rosaceae

**Figure 1:**
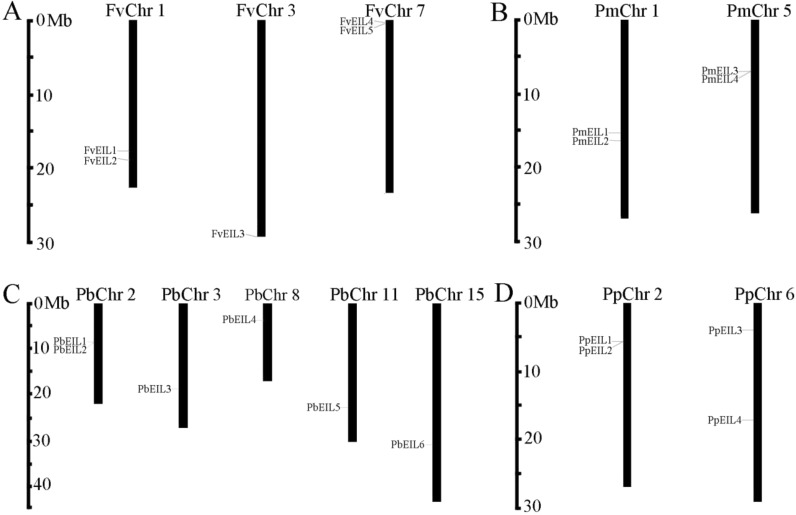
Chromosomal location of *EIN3/EIL* genes in the genomes of strawberry (A), mei (B), pear (C) and peach (D). The distribution of *EIN3/EIL* genes among the chromosomes in each species was diverse. The chromosome number was represented at the top of each chromosome. The left scale indicates the megabases (Mb).

The genome data of pear (*P. bretschneideri*), mei (*P. mume*), peach (*P. persica*) and strawberry (*F. vesca*) were recently published, respectively (Shulaev et al., 2011; Verde et al., 2013; Wu et al., 2012; Zhang et al., 2012). To identify the members of the *EIN3/EIL* gene families in these species, EIN3/EIL specific domain (PF04873) was used to perform Blastp searches of the local protein databases. Sequences identified were verified for EIN3/EIL domains through SMART database and InterPro online tool. In total 24 of *EIN3/EIL* genes were identified, including ten in pear, four in peach, five in mei and five in strawberry, and named as *PbEIL1*-*PbEIL10*, *PpEIL1*-*PpEIL4*, *PmEIL1*-*PmEIL5* and *FvEIL1*-*FvEIL5*, according to their locations in chromosome, respectively (Table 1 and Fig. 1). This result suggested that *EIN3/EIL* gene family was relatively small compared to other gene families in the studied species. Similar indication was also reported by the previous studies in which six, five, four, six and 17 *EIN3/EIL* genes were found in *Arabidopsis thaliana* (Chao et al., 1997; Guo & Ecker, 2004), tobacco (Kosugi & Ohashi, 2000; Rieu, Mariani & Weterings, 2003), tomato (Tieman et al., 2001; Yokotani et al., 2003), rice (Hiraga et al., 2009; Mao et al., 2006) and banana (Jourda et al., 2014), respectively. Furthermore, it was found that the genome sizes and number of *EIN3/EIL* gene family members appeared not to have a direct relevance. For example, although there was no significant variety in genome size of pear (271.9 Mb) and strawberry (240 Mb), the number of *EIN3/EIL* genes obviously changed. Contrarily, the number of *EIN3/EIL* genes of the peach (224.6 Mb) and strawberry (240 Mb) had a corresponding relationship with their genome size. Remarkably, compared with those in peach, mei and strawberry, the numbers of *EIN3/EIL* genes in pear were found to be almost doubled. Moreover, the chromosome numbers of peach, mei and strawberry are 16, 16 and 14, respectively (Shulaev et al., 2011; Verde et al., 2013; Wu et al., 2012; Zhang et al., 2012), whereas the chromosome number of pear is 34, indicating that the *EIN3/EIL* gene family has undergone an expansion corresponding to the variation in chromosome number. However, a recent whole genome duplication event (30–45 million years ago) (Wu et al., 2012) that occurred in pear but not in peach, mei and strawberry probably contributed to the expansion of *EIN3/EIL* gene family in the pear.

**Table 1:** List of *EIN3/EIL* genes identified in pear, peach, mei and strawberry.

Name	Gene model	Chromosme	5′ end	3′ end
*FvEIL1*	mrna25474.1	Chr1	17653671	17655527
*FvEIL2*	mrna16361.1	Chr1	18891616	18892965
*FvEIL3*	mrna20650.1	Chr3	29248944	29253704
*FvEIL4*	mrna00379.1	Chr7	290967	292781
*FvEIL5*	mrna00392.1	Chr7	349495	351202
*PmEIL1*	Pm001950	Chr1	15239829	15241073
*PmEIL2*	Pm002057	Chr1	16248534	16250294
*PmEIL3*	Pm017009	Chr5	6907428	6909233
*PmEIL4*	Pm017011	Chr5	6933006	6934874
*PmEIL5*	Pm028171	scaffold103	1235430	1246520
*PpEIL1*	ppa003493m	Chr2	5516222	5518429
*PpEIL2*	ppa003550m	Chr2	5549949	5552334
*PpEIL3*	ppa003113m	Chr6	3882268	3885188
*PpEIL4*	ppa016118m	Chr6	16979366	16982360
*PbEIL1*	Pbr024739.1	Chr2	8493409	8495211
*PbEIL2*	Pbr024740.1	Chr2	8506285	8508129
*PbEIL3*	Pbr000646.1	Chr3	18718500	18721454
*PbEIL4*	Pbr026603.1	Chr8	3598382	3602224
*PbEIL5*	Pbr004535.1	Chr11	22794386	22798326
*PbEIL6*	Pbr033210.1	Chr15	31009957	31014042
*PbEIL7*	Pbr010447.1	scaffold170.2.1	239188	246113
*PbEIL8*	Pbr010448.1	scaffold170.2.1	259361	262132
*PbEIL9*	Pbr022557.1	scaffold341.0	56672	57973
*PbEIL10*	Pbr039294.1	scaffold837.0	82840	84144

**Notes.**

Pear gene models are found in the GigaDB Genome database; mei and peach gene models are found in the Rosaceae Genome Database; strawberry gene models are found in the Phytozome database.

To determine the distribution of *EIN3/EIL* genes on chromosomes among pear, peach, mei and strawberry, respectively, a chromosome map (Fig. 1) was drawn based on genome annotation (Wu et al., 2012). In pear, two *EIN3/EIL* genes were located on chromosome 2, and one gene on chromosome 3, 8, 11 and 15, respectively, with the remaining genes localized on different scaffold regions (Fig. 1C). In peach, two *EIN3/EIL* genes were found on chromosome 2 and 6, respectively (Fig. 1D). In mei, two *EIN3/EIL* genes were distributed on chromosome 1 and 5, respectively, with the remaining one localized on a scaffold region (Fig. 1B). In strawberry, two *EIN3/EIL* genes were distributed on chromosome 1 and 7, respectively, with the remaining one localized on chromosome 3 (Table 1 and Fig. 1A).

### Phylogenetic analysis of *EIN3/EIL* genes

**Figure 2:**
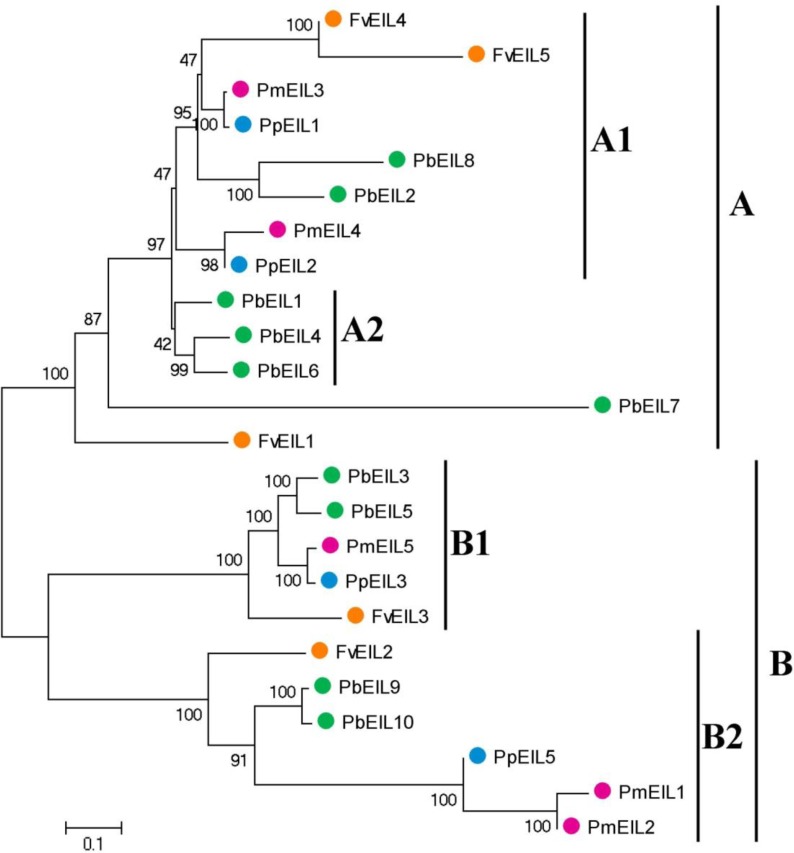
Phylogenetic tree of *EIN3/EIL* proteins from pear, peach, mei and strawberry. The neighbor-joining (NJ) tree was constructed by using MEGA 5 software. The neighbor-joining (NJ) tree was constructed by using MEGA 5 software (1,000 bootstrap replicates). The different colors suggest the different species background for each *EIN3/EIL* protein. Gene names are listed in Table 1. The scale bar represents 0.1 amino acid changes per site.

The phylogenetic tree containing *EIN3/EIL* gene homologs from a variety of species, including *Arabidopsis thaliana*, rice, banana, sorghum, maize, *Brachypodium distachyon*, *Thellungiella parvula*, and four Rosaceae species, was also constructed (Fig S1). As shown in the phylogenetic tree, most *EIN3/EIL* genes from four Rosaceae species were clustered together. To further understand the evolutionary history of *EIN3/EIL* genes in Rosaceae, phylogenetic analysis was carried out using the neighbor joining (NJ) method. As shown in Fig. 2, 24 EIN3/EIL sequences were divided into two subfamilies, designated as A and B, which contained four classes (Classes A1, A2, B1 and B2). Classes A1, B1 and B2 were composed of the *EIN3/EIL* genes from the four species (pear, peach, mei and strawberry), while the Class A2 contained the members only from pear. According to our study, a whole genome duplication event happened 30–45 million years ago in pear, but not in peach, mei and strawberry (Wu et al., 2012). This result suggested the probable reason for occurrence of Class A2 genes in pear. Remarkably, *EIN3/EIL* genes from peach and mei showed higher similarity with each other according to genetic distance, which was consistent with a previous study reporting that the closer relationship between peach and mei versus peach and pear/strawberry (Cao et al., 2016a; Cao et al., 2016b; Cao et al., 2016d).

Each of the four Rosaceae species contributed at least one member of the *EIN3/EIL* gene to each class, with the exception for Class A2 (Fig. 2). Therefore, we deduced that *EIN3/EIL* genes had rapidly been duplicated before these dicotyledon species diverged. However, only the *EIN3/EIL* genes in class A2 revealed an internal duplication. In addition, we identified three pairs of orthologous genes among the *EIN3/EIL* genes: *PmEIL3* and *PpEIL1*, *PpEIL2* and *PmEIL4*, *PpEIL3* and *PmEIL5* based on the phylogenetic analysis.

### Structural analysis of *EIN3/EIL* genes

**Figure 3:**
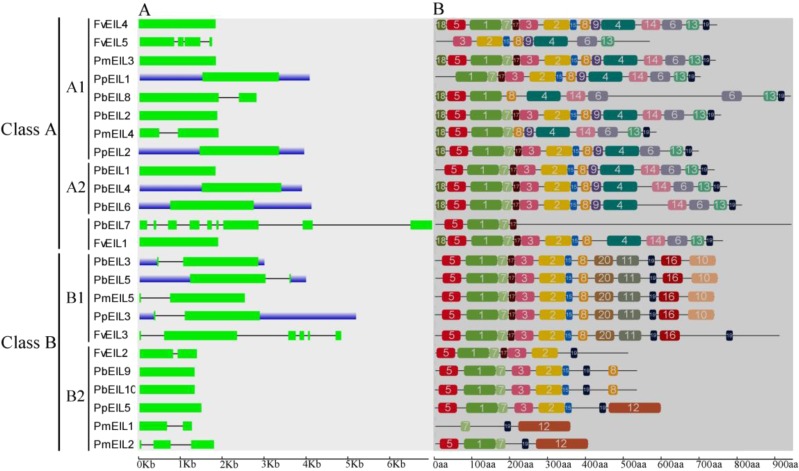
Gene structure (A) and conserved motif compositions (B) of *EIN3/EIL* genes in Rosaceae species. Untranslated regions (UTRs), introns and exons are represented by blue boxes, thin lines and green rectangles, respectively. Note that the gene or protein lengths can be estimated by using the scale at the bottom.

Previous studies have suggested that gene structural diversity is the primary resource for the evolution of multigene families (Cao et al., 2016c; Leitch & Leitch, 2013; Mercereau-Puijalon, Barale & Bischoff, 2002). To characterize the structural diversity of the *EIN3/EIL* gene family, exon-intron organization of each *EIN3/EIL* gene was analyzed. As shown in Fig. 3A, most genes did not contain introns, such as *FvEIL1*, *PbEIL1*, *PmEIL3* and *PpEIL4* et al. Furthermore, *PbEIL7* contained eight introns, followed by *FvEIL3* (five), whereas *FvEIL5* had three introns, *PmEIL2* had two introns and eight *EIN3/EIL* genes contained one intron (Fig. 3A). These results implied that the intron/exon loss and acquire has occurred in the evolution of the *EIN3/EIL* gene family, which may be able to explain the functional divergence of closely related *EIN3/EIL* genes. In the present study, the gene structures of the *EIN3/EIL* homologous gene pairs were investigated. We found that the exon number of two gene pairs (*PbEIL2*/*PbEIL8* and *PmEIL1*/*PmEIL2*) had changed. Further analysis indicated that *PbEIL8* and *PmEIL2* obtained one exon during evolution, while *PbEIL2* and *PmEIL1* lost one exon. These diversities might be due to single intron loss or obtain events during evolution.

Because 24 *EIN3/EIL* genes did not have high similarity, MEME web server was used to find conserved motifs. Subsequently, we identified 20 conserved motifs, which were shown in Table S2 and Fig. 3B. The Pfam and SMART databases were used to annotate the individual of the putative motifs. Motif 1 and motif 2 were identified to encode a conserved EIN3/EIL domain (Chang & Shockey, 1999; Chao et al., 1997), whereas the remaining motifs did not get function annotation. Most EIN3/EIL proteins have motifs 1, 2, 4, 7, 10 and 19. In addition, several proteins from clade B2 contained unique motif 12, which might imply its specific functions. Remarkably, most of the closely related EIN3/EIL proteins in the same clade exhibited similar motif compositions (e.g., PmEIL5/PpEIL3 and PbEIL3/PbEIL5) indicating their functional similarity among these EIN3/EIL proteins. Summarily, the similarity in motif distribution and exon-intron structure of most EIN3/EIL proteins supported the results from phylogenetic analysis of the *EIN3/EIL* genes, whereas the differences of the related characteristics in the different classes indicated that their functions were diversified.

### Conserved microsynteny of *EIN3/EIL* genes in the four Rosaceae species

Based on the whole-genome data, species microsynteny can be used to identify the location of orthologous genes and/or paralogous genes (Cao et al., 2016d; Lin et al., 2014). To identify the homologous genes (orthology or paralogy) within the *EIN3/EIL* genes from four Rosaceae species (pear, peach, mei and strawberry), as well as their evolutionary history, microsynteny analysis was performed. By pairwise comparisons of flanking sequences in the chromosomal regions containing *EIN3/EIL* genes, three or more pairs were present in this region, which were considered as either conserved microsynteny or high levels of microsynteny.

**Figure 4:**
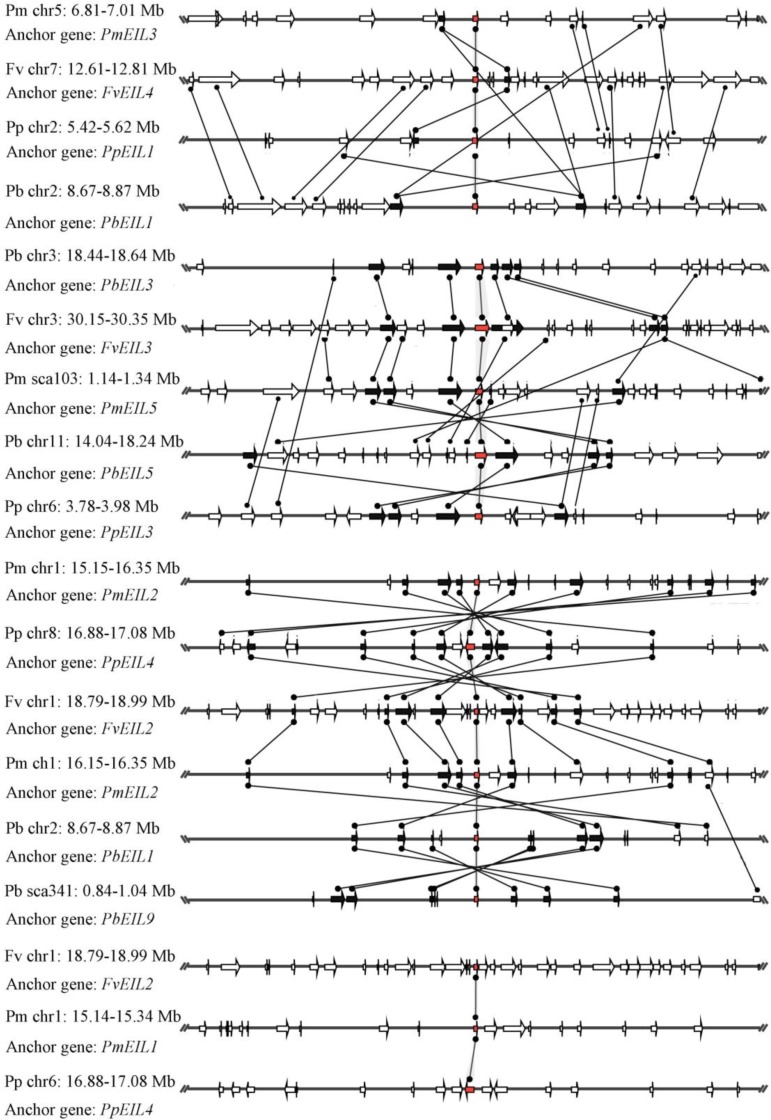
Interspecies microsynteny related to *EIN3/EIL* families in four Rosaceae. The relative positions of all flanking protein-coding genes were defined by anchored *EIN3/EIL* genes, highlighted in red. The gene’s orientations are shown as triangle, with gray lines corresponding to chromosomal segments.

**Figure 5:**
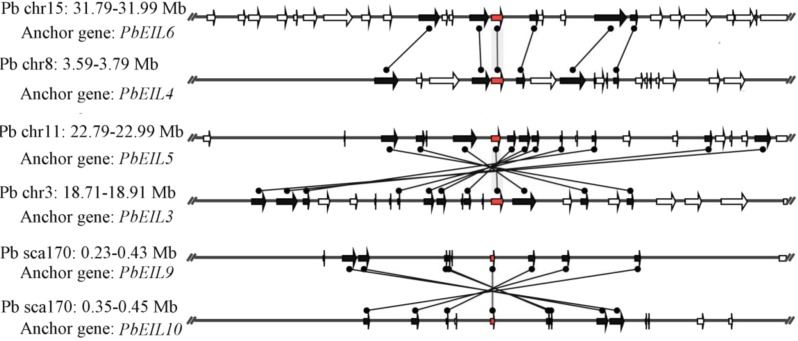
Intraspecific microsynteny related to *EIN3/EIL* families with the same species. The relative positions of all flanking protein-coding genes were defined by anchored *EIN3/EIL* genes, highlighted in red. The gene’s orientations are shown as triangle, with gray lines corresponding to chromosomal segments.

In this study, a total of 55 flanking sequences containing *EIN3/EIL* genes could be assembled into 28 regions and divided into seven microsynteny groups. It was supposed that *EIN3/EIL* genes from the same group should evolve from the most recent common ancestor. Based on this criterion, orthology and/or paralogy relationships, as well as their evolutionary origins, were detected among *EIN3/EIL* genes of the four Rosaceae species. Nine, five, four and four out of the seven microsynteny groups are from pear, peach, mei and strawberry, respectively (Fig. 4). In class B2, two gene pairs (*PbEIL1* and *PbEIL9*, *PbEIL9* and *PbEIL10*) with both a higher level of microsynteny and a noticeable inverted duplication, were identified. Interestingly, it was also found that some duplication rules in several regions were in disorder, such as *FvEIL1* and *FvEIL2*, *FvEIL1* and *PpEIL4* (Fig. 4). Similar microsynteny was identified in other classes with a concordant inverted microsynteny (Fig. 4). In addition, according to the constructed phylogenetic tree, conservation of microsynteny between different families appeared gradually. However, some flanking genes in each microsyntenic group were not conserved, indicating that they arose later than this duplication event (Fig. 4). Furthermore, we only identified four pairs of intraspecies microsynteny groups from pear (*PbEIL1* and *PbEIL9*, *PbEIL3* and *PbEIL5*, *PbEIL4* and *PbEIL6*, *PbEIL9* and *PbEIL10*), but peach, mei and strawberry were excluded (Fig. 5). This difference might result from the expansion of pear *EIN3/EIL* genes. However, no similar gene expansion was identified in peach, mei and strawberry. Some previous studies has hypothesized that transcription factors should be generally and preferentially retained after genome duplications (Blanc & Wolfe, 2004), with a lower frequency of tandem duplication events in a number of transcription factors (Freeling, 2009). Additionally, genes from whole-genome duplication events are more easily retained into genomes. With the stoichiometric relationships, these genes were strongly retained by stabilizing selection (Lynch & Conery, 2000). Our results were not only consistent with this hypothesis, but was also strong evidence for it.

**Table 2:** The relative syntenic quality of *EIN3/EIL* genes in four Rosaceae plants.

	Clade A1	Clade A2	Clade B1	Clade B2	Average
Pb-Pp			22.50%	44.07%	33.29%
Pb-Pm			21.51%	41.18%	31.35%
Pb-Fv			24.07%	28.26%	26.17%
Pp-Pm	26.67%		40.00%	29.73%	32.13%
Pp-Fv	10.26%				10.26%
Pm-Fv	10.00%		26.32%	3.77%	13.36%
					24.43%

**Notes.**

The relative syntenic quality was estimated as twice the number of matches divided by the sum of the total number of genes in both conserved gene regions, based on the previous methods (Cannon et al., 2003; Cannon et al., 2006).

Subsequently, the quality of the synteny was estimated in four Rosaceae plants based on previous research methods (Cannon et al., 2003; Cannon et al., 2006). As shown in Table 2, the relative synteny quality of the *EIN3/EIL* genes from these Rosaceae four species genomes was 24.43% for orthologous regions. The highest value of synteny quality found between pear and peach was 33.29%. And the lower value of synteny quality was obtained between strawberry and peach (10.26%) and mei (13.26%) The relative synteny quality in the pear/mei and pear/strawberry syntenic regions was 31.35% and 26.17%, which was substantially lower than the 32.13% found in the pear/peach synteny blocks. Our results were essentially consistent with their evolutionary relationship (Xiang et al., 2017; Zhong et al., 2015).

### Strong purifying selection for *EIN3/EIL* genes in four Rosaceae species

In general, Ks values can be used to estimate evolutionary data of the whole genome duplication events or segmental duplication events. Previous studies showed that pear had experienced two whole genome duplication events, including an ancient whole genome duplication (Ks ∼ 1.5–1.8) estimated at ∼140 MYA (Fawcett, Maere & Van de Peer, 2009) and a recent whole genome duplication (Ks ∼ 0.15–0.3) estimated at 30–45 MYA (Wu et al., 2012), while peach, mei and strawberry only experienced an ancient whole genome duplication event. Therefore, Ks values were applied to analyze the whole genome duplication or segmental duplication events in *EIN3/EILs* of four Rosaceae species. As shown in Table S3, the mean Ks values of each duplication pairs in the syntenic region were lists. In pear, we found the mean Ks values of *EIN3/EIL* gene pairs were 0.0363, 0.1717 and 0.2836, respectively. It was obvious that these duplications might be resulting from the latest whole genome duplication (30–45 MYA; Ks ∼ 0.15–0.3), but an ancient whole genome duplication (∼140MYA; Ks ∼ 1.5–1.8) in pear.

**Figure 6:**
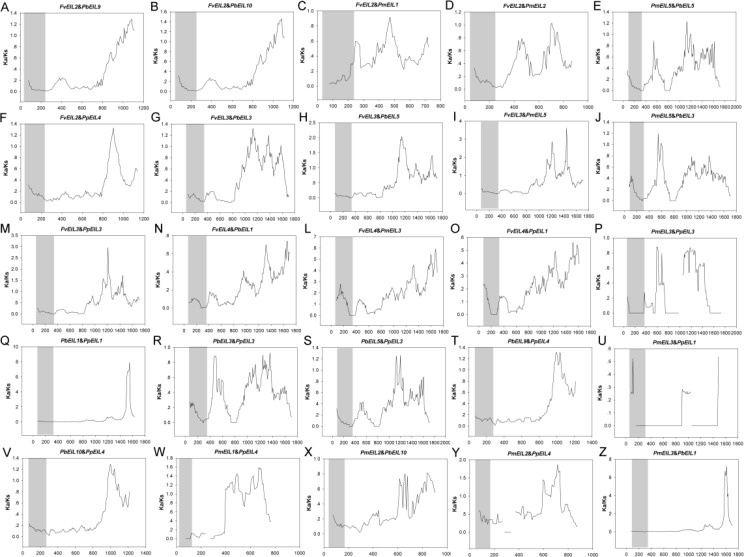
Sliding window plots of duplicated *EIN3/EIL* genes in Rosaceae species. The gray blocks indicate the positions of the *EIN3/EIL* domains. The window size is 150 bp, and the step size is 9 bp. The *x*-axis denotes the synonymous distances within each gene.

In addition, the Ka/Ks values are widely used to represent the gene selection pressure and evolution rate [40]: Ka/Ks value with >1 indicates positive selection with accelerated evolution, Ka/Ks < 1 indicates negative/purifying selection with the functional constraint, and Ka/Ks = 1 suggests that the genes are drifting neutrally. In this study, all paralogs was found with Ka/Ks ratios <1 (Fig. 6), indicating their purifying selection. Furthermore, to better understand the delineate regions of diversifying and purifying selection in the *EIN3/EIL* gene family, a sliding window analysis of the Ka/Ks values between paralogs was performed (Fig. 4); the EIN3/EIL domains in the N-termini exhibited stronger purifying selection compared with the whole gene regions (C-termini). These results suggested that the *EIN3/EIL* genes had undergone strongly purifying selection, especially for EIN3/EIL domains in the N-termini (Fig. 4Q). Overall, strong evolutionary constraints were involved in *EIN3/EIL* gene evolution, which may contribute to their functional stability. On the other hand, some parts of protein-coding genes had undergone positive selection, implying the generation of innovative gene functions.

**Figure 7:**
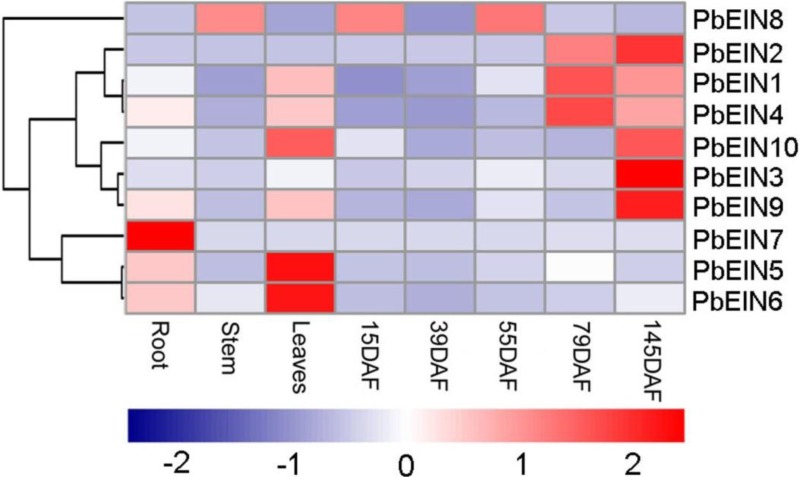
Expression profiling of pear *PbEIL* genes in eight samples from root, stem, leaves and fruits in several development stages. The expression profile data was obtained with qRT-PCR experiments. Blue and red colors indicate low-expression and high-expression, respectively.

### Expression profiles analysis of *PbEIL* genes in different tissues

To increase our understanding of the potential functions of pear *EIN3/EIL* genes during development, qRT-PCR analysis was carried out to determine the expression profiles of ten *PbEIL* genes in different tissues. As shown in Fig. 7 and Table S4, ten pear *EIN3/EIL* genes showed significantly different tissue-specific expression patterns in eight samples from root, stem, leaves and fruits in several development stages. Among the ten pear *EIN3/EIL* genes, three (*PbEIL5*, *PbEIL6* and *PbEIL10*) showed the highest transcript accumulation in the leaves, three (*PbEIL2*, *PbEIL3* and *PbEIL9*) in 145 DAF (days after flowering), two (*PbEIL1* and *PbEIL4*) in 79 DAF, and one (*PbEIL7*) in the roots. Additionally, the duplication gene pairs showed different expression patterns; for example, *PbEIL4* was highly expressed in 79 DAF, while its duplication gene, *PbEIL6*, was expressed at a high level in the leaves. Thus, the pear *EIN3/EIL* duplicates resulting from recent whole genome duplication have different expression patterns in several different tissues, indicating subfunctionalization after duplication. At the same time, this phenomenon was also observed among other *EIN3/EIL* duplication genes (Jourda et al., 2014).

## Conclusions

In this study, we identified 24 *EIN3/EIL* genes from four Rosaceae species (pear, peach, mei and strawberry). Subsequently, a systematic analysis, including their chromosomal location, evolutionary relationship, conserved microsynteny, gene structure and sliding window, was carried out. According to phylogenetic analysis, the *EIN3/EIL* genes divided into four classes. Remarkably, high level of microsynteny of the *EIL/EIN3* family in Rosaceae was found, indicating that the genome duplication plays a key role in the expansion of the *EIL/EIN3* genes in the Rosaceae. In these *EIL/EIN3* genes, all paralogs have experienced purifying selection, especially the EIL/EIN3 domains in the Rosaceae. Furthermore, the expression profiles of the *PbEIL* genes suggested that the recent whole genome duplication derived genes show indications of subfunctionalization. These results may help promote the extrapolation of *EIL/EIN3* gene functions in future.

##  Supplemental Information

10.7717/peerj.3400/supp-1 Supplemental Information 1 Supplemental files

10.7717/peerj.3400/supp-2 Supplemental Information 2 List of *EIN3/EIL* genes identified in pear, peach, yangmei and strawberry

## Additional Information and Declarations

### Data Availability

The following information was supplied regarding data availability: The raw data has been supplied as Supplementary File.
