# Supplementary material for: Genome-wide analysis suggests high level of microsynteny and purifying selection affect the evolution of EIN3/EIL family in Rosaceae
Source: PeerJ. 2017 May 31;5:e3400. doi: 10.7717/peerj.3400 (PMC5455322; doi:10.7717/peerj.3400)
Supplement: Supplemental Information 1 [file peerj-05-3400-s001.docx]

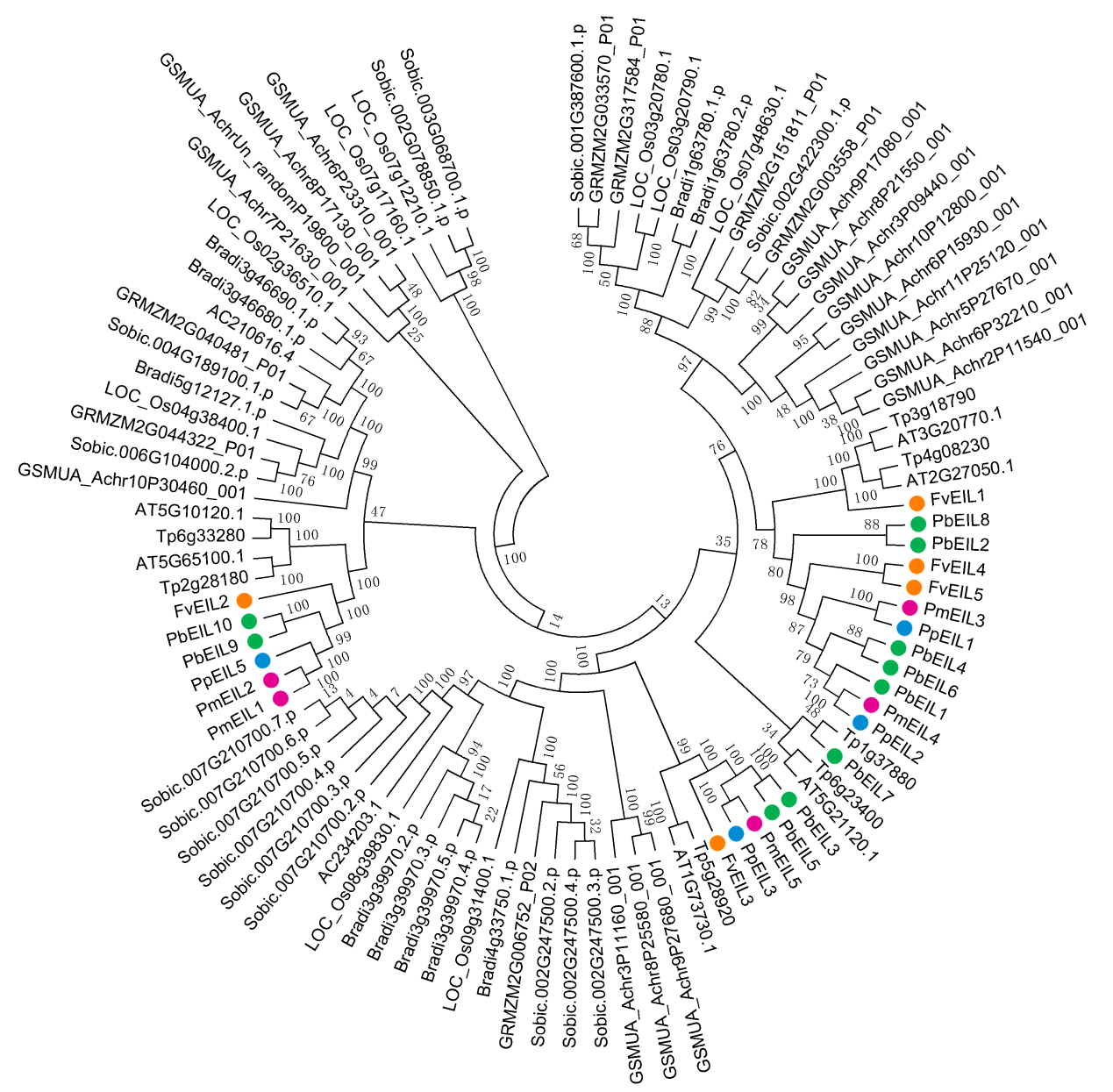


**Figure S1.** Phylogenetic analysis of the *EIN3/EIL* gene family. The amino acid sequences of *EIN3/EIL* genes were aligned by using ClustalX version 1.83 and the neighbor-joining tree was generated by using MEGA 5 software (1000 bootstrap replicates). The *EIN3/EIL* genes of *Thellungiella parvula* (Tp), *Brachypodium distachyon* (Bradi), Arabidopsis (At), rice (LOC), maize (GRMZM or AC), banana (GSMUA) and sorghum (Sobic) were obtained from Plant Transcription Factor Databases ([Jin et al. 2017](#_ENREF_1)).

| **Table S1**. qRT-PCR primers for *PbEIL* genes expression profile | |
| --- | --- |
| ID | Primer |
| PbEIL1-F | CGTCAACATGAGATTCGACTC |
| PbEIL1-R | CTTGCTTTGGCGGAGTGT |
| PbEIL2-F | TAATCTTCCCGATAATACTGAC |
| PbEIL2-R | CAACGACACATCCAGAAT |
| PbEIL3-F | GTCATTAGATTATGGATTATTCAA |
| PbEIL3-R | CATCATTTCTTCGAGTTCA |
| PbEIL4-F | CCCCTTTCACTTGGCACC |
| PbEIL4-R | ATGGGAACCAGGGAGATG |
| PbEIL5-F | TGTCGTTGGATTATGGAT |
| PbEIL5-R | CGAGTTCAAGTTCATCTAAT |
| PbEIL6-F | GTAACAATTCCAACGAAGATG |
| PbEIL6-R | GGCGGGTCCATAACATTA |
| PbEIL7-F | AGAAGAGGTAGCATCTAT |
| PbEIL7-R | TGAACTCATCAAAGGTAA |
| PbEIL8-F | ACTTATTTGATTATCCATTTGGTA |
| PbEIL8-R | AGCAGCAAGTCAACATTA |
| PbEIL9-F | ACAAGTTGATCCTCTAAG |
| PbEIL9-R | AATACTATCCTTCCACATAC |
| PbEIL10-F | GGAAACAAGGAAATAAATCGTTCT |
| PbEIL10-R | CCAAATCCCAAATAGAAGTTGAAT |
| Note: F and R represent 5'Primer and 3'Primer, respectively | |

| **Table S2**. All MEME motif sequences in Rosaceae EIN3/EIL proteins. | | |
| --- | --- | --- |
| Motif | Best possible match | Best possible match |
| 1 | 69 | DNARQRQSQEQARRKKMSRAQDGILKYMLKMMEVCKAQGFVYGIIPEKGKPVTGASDNLRAWWKEKVRF |
| 2 | 57 | PPYKKPHDLKKAWKVGVLTAVIKHMSPDIAKIRRHVRQSKCLQDKMTAKESATWLAI |
| 3 | 41 | DTTLGSLLSALMQHCDPPQRRFPLEKGVAPPWWPTGNEEWW |
| 4 | 74 | PQIKGELIETNSDFGQKRKQLAEEPQMMLNQKIYTCEYPQCPYHDYRLGFLDITARNNHQLNCAYRNNSSQVFG |
| 5 | 41 | AAPEHECEATVEEDYSDEEIDYDELEKRMWRDRMRLKRLKE |
| 6 | 44 | NIQQNKNCNPGNLNVVEDHNQQQVNYQFPMNDNFYGQGVVMGRN |
| 7 | 21 | DRNGPAAISKYQAEHIIPGKN |
| 8 | 21 | GSGSFAISGTSDYDVEGVDDE |
| 9 | 21 | VEIEDCKPHVNHFNIGTAGQR |
| 10 | 57 | DTFQSEQDRTVNANFGSPIDSLSLDYGLFNSPFHFGIDGTGSLDDLELEEMMEYFAA |
| 11 | 49 | NIHLGPRNALPDINHTDVQMIGFQVHENQQENGTITTLRPPENDLDVQA |
| 12 | 113 | SGLLAEGKFPLFDEFPIDTGVMSMVDWINMELQKANQIDQNSGAFVINGQVGGEVSDTTVGDYGASYWGGGIEDLAIDGAFDIQRGNMDLNLCSEEEISHNQESTSIWDLGYD |
| 13 | 29 | PMHHPVFPSTEVQFDQCKVFDSPFGNNPN |
| 14 | 41 | FSLPITQPKPAIQQPVNQTSSFNASGLGLAEDGQKMISELM |
| 15 | 15 | INQEEALIRRLYPDR |
| 16 | 44 | EVHHGDTFNLYNPSAEYPPSHDQQPSQIVMNEPQIRPADGVHIP |
| 17 | 15 | CSAVASTPHTLQELQ |
| 18 | 21 | MGIFEEMGFCGNLDFLSAPPG |
| 19 | 15 | LTFGFHTHLAPVDYH |
| 20 | 41 | PMDVEPSSNLCNNTRNHVQDKEQGEKKPRRKRPRVRASPVE |

| **Table S3**. Estimates of ages for the large-scale genome duplication events in four Rosaceae species. | |
| --- | --- |
| Duplicated *EIN3/EIL* gene pairs | Number of conserved flanking protein-coding genes |
| FvEIL4 and PbEIL1 | 8 |
| FvEIL4 and PmEIL3 | 1 |
| FvEIL4 and PpEIL1 | 1 |
| FvEIL2 and PpEIL4 | 8 |
| FvEIL2 and PmEIL1 | 0 |
| FvEIL2 and PmEIL2 | 9 |
| FvEIL2 and PbEIL9 | 5 |
| FvEIL2 and PbEIL10 | 5 |
| FvEIL3 and PpEIL3 | 3 |
| FvEIL3 and PmEIL5 | 5 |
| FvEIL3 and PbEIL5 | 6 |
| FvEIL3 and PbEIL3 | 3 |
| PmEIL1 and PpEIL4 | 0 |
| PmEIL2 and PpEIL4 | 9 |
| PmEIL2 and PbEIL10 | 6 |
| PmEIL3 and PpEIL1 | 3 |
| PmEIL5 and PpEIL3 | 5 |
| PmEIL3 and PbEIL1 | 7 |
| PmEIL5 and PbEIL5 | 6 |
| PmEIL5 and PbEIL3 | 5 |
| PbEIL3 and PbEIL5 | 11 |
| PbEIL3 and PpEIL3 | 7 |
| PbEIL5 and PpEIL3 | 6 |
| PbEIL9 and PbEIL10 | 7 |
| PbEIL9 and PpEIL4 | 4 |
| PbEIL1 and PpEIL1 | 6 |
| PbEIL4 and PbEIL6 | 6 |
| PbEIL10 and PpEIL4 | 4 |
| Note: s.d.: standard deviation | |

**Table S4**. Expression of *PbEIL* genes in eight samples from root, stem, leaves and fruits in several development stage.

| ID | Root | Stem | Leaves | 15DAF | 39DAF | 55DAF | 79DAF | 145DAF |  |
| --- | --- | --- | --- | --- | --- | --- | --- | --- | --- |
| *PbEIN1* | 1 | 0.3383684 | 1.6170153 | 0.2111978 | 0.3056601 | 0.8888427 | 2.5082248 | 1.977028 |  |
| *PbEIN2* | 1 | 0.011023 | 0.0450187 | 6.5432165 | 10.314962 | 4.0934956 | 372.21696 | 528.83237 |  |
| *PbEIN3* | 1 | 0.3551909 | 1.7735828 | 0.0098431 | 0.4579726 | 1.6358041 | 0.7136721 | 13.547925 |  |
| *PbEIN4* | 1 | 0.2448551 | 1.2397077 | 0.1116199 | 0.0595399 | 0.3120826 | 2.2345743 | 1.5439935 |  |
| *PbEIN5* | 1 | 0.3360311 | 2.0514822 | 0.3568361 | 0.3253355 | 0.4622248 | 0.6814438 | 0.4263174 |  |
| *PbEIN6* | 1 | 0.4954003 | 2.2294173 | 0.2151379 | 0.1166291 | 0.2471285 | 0.3171711 | 0.5260724 |  |
| *PbEIN7* | 1 | 0.0002753 | 0.0018953 | 0.0006503 | 6.587E-05 | 0.0032772 | 0.0076341 | 0.0073062 |  |
| *PbEIN8* | 1 | 1.7330741 | 0.8766057 | 1.737083 | 0.8066418 | 1.8025009 | 1.0163049 | 0.9614831 |  |
| *PbEIN9* | 1 | 0.2171354 | 1.2541124 | 0.1303082 | 0.0774817 | 0.5321851 | 0.2819152 | 2.6390158 |  |
| *PbEIN10* | 1 | 0.4033209 | 3.2116915 | 0.7791646 | 0.0938608 | 0.3275983 | 0.2068514 | 3.279176 |  |
| Note: DAF: days after flowering | | |  |  |  |  |  |  |  |

**References**

Jin J, Tian F, Yang DC, Meng YQ, Kong L, Luo J, and Gao G. 2017. PlantTFDB 4.0: toward a central hub for transcription factors and regulatory interactions in plants. *Nucleic acids research* 45:D1040-D1045.
