# Supplementary material for: Genome-wide analysis suggests high level of microsynteny and purifying selection affect the evolution of EIN3/EIL family in Rosaceae
Source: PeerJ. 2017 May 31;5:e3400. doi: 10.7717/peerj.3400 (PMC5455322; doi:10.7717/peerj.3400)
Supplement: Supplemental Information 2 [file peerj-05-3400-s002.docx]

**Table 1**. List of EIN3/EIL genes identified in pear, peach, yangmei and strawberry.

| **Name** | **Gene Model** | **Chromosme** | **5' End** | **3' End** |
| --- | --- | --- | --- | --- |
| *FvEIL1* | mrna25474.1 | [Chr1](http://www.rosaceae.org/gb/gbrowse/fragaria_vesca_v1.0-lg?name=LG1:17653671..17655527&enable=NCBI%20Sequence%20Alignments) | 17653671 | 17655527 |
| *FvEIL2* | mrna16361.1 | Chr1 | 18891616 | 18892965 |
| *FvEIL3* | mrna20650.1 | [Chr3](http://www.rosaceae.org/gb/gbrowse/fragaria_vesca_v1.0-lg?name=LG3:29248944..29253704&enable=NCBI%20Sequence%20Alignments) | 29248944 | 29253704 |
| *FvEIL4* | mrna00379.1 | [Chr7](http://www.rosaceae.org/gb/gbrowse/fragaria_vesca_v1.0-lg?name=LG7:290967..292781&enable=NCBI%20Sequence%20Alignments) | 290967 | 292781 |
| *FvEIL5* | mrna00392.1 | [Chr7](http://www.rosaceae.org/gb/gbrowse/fragaria_vesca_v1.0-lg?name=LG7:349495..351202&enable=NCBI%20Sequence%20Alignments) | 349495 | 351202 |
| *PmEIL1* | Pm001950 | Chr1 | 15239829 | 15241073 |
| *PmEIL2* | Pm002057 | Chr1 | 16248534 | 16250294 |
| *PmEIL3* | Pm017009 | Chr5 | 6907428 | 6909233 |
| *PmEIL4* | Pm017011 | Chr5 | 6933006 | 6934874 |
| *PmEIL5* | Pm028171 | scaffold103 | 1235430 | 1246520 |
| *PpEIL1* | ppa003493m | Chr2 | 5516222 | 5518429 |
| *PpEIL2* | ppa003550m | Chr2 | 5549949 | 5552334 |
| *PpEIL3* | ppa003113m | Chr6 | 3882268 | 3885188 |
| *PpEIL4* | ppa016118m | Chr6 | 16979366 | 16982360 |
| *PbEIL1* | Pbr024739.1 | Chr2 | 8493409 | 8495211 |
| *PbEIL2* | Pbr024740.1 | Chr2 | 8506285 | 8508129 |
| *PbEIL3* | Pbr000646.1 | Chr3 | 18718500 | 18721454 |
| *PbEIL4* | Pbr026603.1 | Chr8 | 3598382 | 3602224 |
| *PbEIL5* | Pbr004535.1 | Chr11 | 22794386 | 22798326 |
| *PbEIL6* | Pbr033210.1 | Chr15 | 31009957 | 31014042 |
| *PbEIL7* | Pbr010447.1 | scaffold170.2.1 | 239188 | 246113 |
| *PbEIL8* | Pbr010448.1 | scaffold170.2.1 | 259361 | 262132 |
| *PbEIL9* | Pbr022557.1 | scaffold341.0 | 56672 | 57973 |
| *PbEIL10* | Pbr039294.1 | scaffold837.0 | 82840 | 84144 |

**Note:** Pear gene models are found in the GigaDB Genome database; yangmei and peach gene models are found in the Rosaceae Genome Database; strawberry gene models are found in the Phytozome database.
